# Population genetic structure of the deep‐sea mussel *Bathymodiolus platifron*s (Bivalvia: Mytilidae) in the Northwest Pacific

**DOI:** 10.1111/eva.12696

**Published:** 2018-10-12

**Authors:** Ting Xu, Jin Sun, Hiromi K. Watanabe, Chong Chen, Masako Nakamura, Rubao Ji, Dong Feng, Jia Lv, Shi Wang, Zhenmin Bao, Pei‐Yuan Qian, Jian‐Wen Qiu

**Affiliations:** ^1^ Department of Biology Hong Kong Baptist University Hong Kong China; ^2^ Department of Ocean Science Hong Kong University of Science and Technology Hong Kong China; ^3^ Japan Agency for Marine‐Earth Science and Technology (JAMSTEC) Yokosuka Japan; ^4^ School of Marine Science and Technology Tokai University Shizuoka Japan; ^5^ Department of Biology Woods Hole Oceanographic Institution Woods Hole Massachusetts; ^6^ CAS Key Laboratory of Ocean and Marginal Sea Geology South China Sea Institute of Oceanology Chinese Academy of Sciences Guangzhou China; ^7^ Ministry of Education Key Laboratory of Marine Genetics and Breeding College of Marine Life Sciences Ocean University of China Qingdao China; ^8^ Laboratory for Marine Biology and Biotechnology Qingdao National Laboratory for Marine Science and Technology Qingdao China; ^9^ Laboratory for Marine Fisheries Science and Food Production Processes Qingdao National Laboratory for Marine Science and Technology Qingdao China

**Keywords:** *Bathymodiolus*, deep‐sea, genetic structure, introgression, migration patterns, mitochondrial genes, population connectivity, RAD‐seq

## Abstract

Studying population genetics of deep‐sea animals helps us understand their history of habitat colonization and population divergence. Here, we report a population genetic study of the deep‐sea mussel *Bathymodiolus platifrons* (Bivalvia: Mytilidae) widely distributed in chemosynthesis‐based ecosystems in the Northwest Pacific. Three mitochondrial genes (i.e., *atp6*,* cox1*, and *nad4*) and 6,398 genomewide single nucleotide polymorphisms (SNPs) were obtained from 110 individuals from four hydrothermal vents and two methane seeps. When using the three mitochondrial genes, nearly no genetic differentiation was detected for *B. platifrons* in the Northwest Pacific. Nevertheless, when using SNP datasets, all individuals in the South China Sea (SCS) and three individuals in Sagami Bay (SB) together formed one genetic cluster that was distinct from the remaining individuals. Such genetic divergence indicated a genetic barrier to gene flow between the SCS and the open Northwest Pacific, resulting in the co‐occurrence of two cryptic semi‐isolated lineages. When using 125 outlier SNPs identified focusing on individuals in the Okinawa Trough (OT) and SB, a minor genetic subdivision was detected between individuals in the southern OT (S‐OT) and those in the middle OT (M‐OT) and SB. This result indicated that, although under the influence of the Kuroshio Current and the North Pacific Intermediate Water, subtle geographic barriers may exist between the S‐OT and the M‐OT. Introgression analyses based on these outlier SNPs revealed that Hatoma Knoll in the S‐OT represents a possible contact zone for individuals in the OT‐SB region. Furthermore, migration dynamic analyses uncovered stronger gene flow from Dai‐yon Yonaguni Knoll in the S‐OT to the other local populations, compared to the reverse directions. Taken together, the present study offered novel perspectives on the genetic connectivity of *B. platifrons* mussels, revealing the potential interaction of ocean currents and geographic barriers with adaption and reproductive isolation in shaping their migration patterns and genetic differentiation in the Northwest Pacific.

## 1 INTRODUCTION

Hydrothermal vents and cold seeps generally occur in tectonically active areas and along continental margins, where neighboring sites are often separated by tens to hundreds of kilometers in the ocean (Le Bris et al., 2016). Despite differences in water temperature and main source of fluid, vent and seep ecosystems are both fueled mainly by chemosynthesis, the conversion of carbon dioxide and/or methane into organic matters in microbes via oxidation of reduced substances, such as hydrogen sulfide, methane, and hydrogen, unlike shallow‐water ecosystems that are driven primarily by photosynthesis (Tunnicliffe, Juniper, & Sibuet, 2003). High chemosynthetic primary production enables these ecosystems to support a much higher biomass and abundance of megafauna compared to the surrounding seabed (Levin et al., 2016).

Most marine benthic animals, including those in the deep ocean, have a biphasic life with a pelagic larval stage through which they achieve connectivity across different habitats (Cowen & Sponaugle, 2009). Knowledge on population connectivity of vent and seep animals sheds light on the scale, direction, and frequency of dispersal, which will not only enhance our understanding of the mechanisms shaping their global and regional biogeography, but also provide key insights into their recovery potential in response to environmental and anthropogenic disturbances (Baco et al., 2016; Kinlan & Gaines, 2003; Miller, Thompson, Johnston, & Santillo, 2018; Rogers et al., 2012).

**Figure 1:**
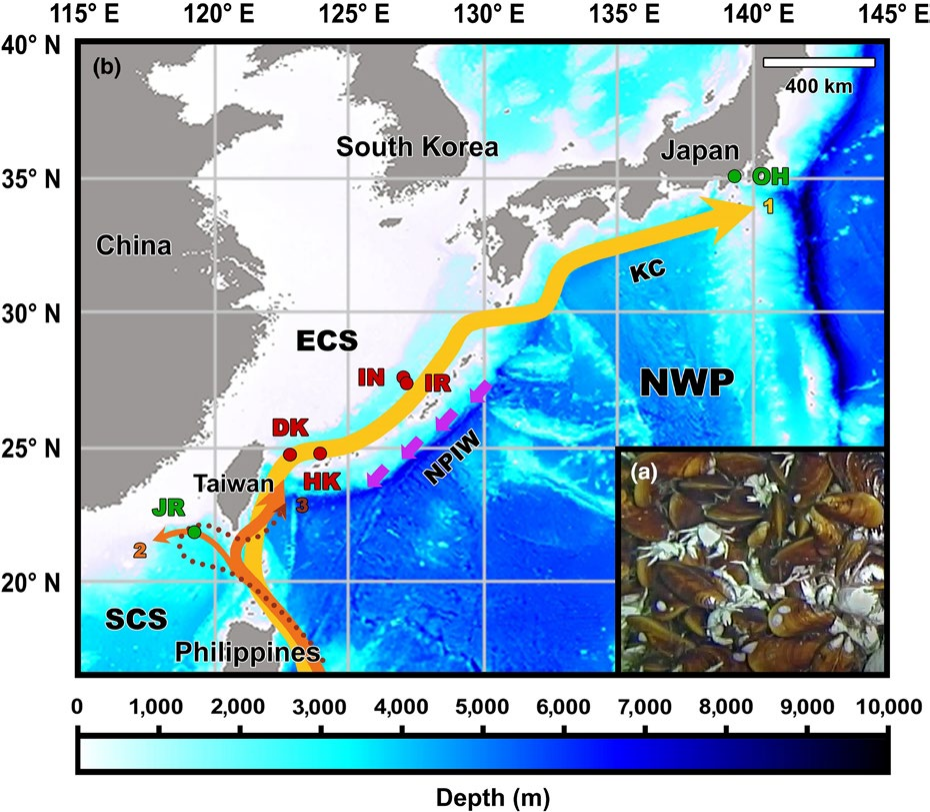
Distribution of *B. platifrons* in the Northwest Pacific. (a) A representative photograph of JR showing the *B. platifrons* and *Shinkaia crosnieri* (squat lobster) dominated community. (b) Sampling locations of *B. platifrons* and the dominant ocean currents in the study region. Vents and seeps are represented in red and green dots, respectively. Ocean currents were redrawn based on You et al. (2005) and Nan et al. (2011). Three flow patterns of the Kuroshio Current when passing through the Luzon Strait, namely the leaping, the leaking, and the looping path, are indicated by 1, 2, and 3, respectively. Code: DK, Dai‐yon Yonaguni Knoll; ECS, East China Sea; HK, Hatoma Knoll; IN, Iheya North; IR, Iheya Ridge; JR, Jiaolong Ridge; KC, Kuroshio Current; NPIW, North Pacific Intermediate Water; NWP, Northwest Pacific; OH, Off Hatsushima; SCS, South China Sea

Deep‐sea mussels in the genus *Bathymodiolus* (Bivalvia: Mytilidae) are one of the most iconic, dominant, and important foundation taxa in chemosynthesis‐based ecosystems (Van Dover, 2000). Dense *Bathymodiolus* mussel beds generate a highly complex habitat for a variety of other animals to inhabit (Figure 1a; Bruno & Bertness, 2001; Govenar, 2010; Vrijenhoek, 2010). *Bathymodiolus* mussels produce planktotrophic larvae capable of migrating to surface water and dispersing across a long distance in ocean currents with very long planktonic larval durations (Arellano, Van Gaest, Johnson, Vrijenhoek, & Young, 2014; McVeigh, Eggleston, Todd, Young, & He, 2017; Young et al., 2012). To date, 30 species including eight fossil species of *Bathymodiolus* have been reported (MolluscaBase, 2018), although the genus appears to be polyphyletic (Lorion et al., 2014). Among them, mussels of the Northwest Pacific species *Bathymodiolus platifrons* are considered to be a good candidate for population genetic studies due to their wide horizontal (22° to 35°N) and bathymetric (642 to 1,684 m) distribution ranges, as well as their capability to inhabit both hydrothermal vents and methane seeps (Fujikura et al., 2007; Suess, 2005; Watanabe, Fujikura, Kojima, Miyazaki, & Fujiwara, 2010; http://www.godac.jamstec.go.jp/bio-sample/index_e.html, April 2018).

Previous studies based on one or several mitochondrial genes revealed a lack of genetic differentiation among vent and seep populations of *B. platifrons* (Kyuno et al., 2009; Miyazaki et al., 2013; Shen et al., 2016). Nevertheless, our recent study based on 9,307 genomewide single nucleotide polymorphisms (SNPs) generated by the type IIB restriction site‐associated DNA (2b‐RAD) approach detected a clear genetic divergence between individuals of *B. platifrons* from a vent field in the Okinawa Trough (OT) and a methane seep in the South China Sea (SCS) (Xu et al., 2017). However, due to the difference in results based on different genetic marker types and limited sampling locations in these studies, the population structure and migration patterns of *B. platifron*s in the Northwest Pacific remain puzzling.

Therefore, we carried out a population genetic study of *B. platifrons* combining both mitochondrial genes and genomewide SNP markers derived from samples from all representative habitats of this species known thus far. Through this study, we aimed to better understand the patterns and causes of genetic connectivity, genetic divergence, and migration dynamics of *B. platifrons*, the most widely distributed *Bathymodiolus* mussel in the Northwest Pacific.

## 2 MATERIALS AND METHODS

### 2.1 Sample collection and DNA extraction

A total of 110 adults of *B. platifrons* used in this study were collected from four hydrothermal vents and two methane seeps between 2009 and 2014 (Figure 1b), either by the remotely operated vehicle (ROV) *Hyper‐Dolphin* on‐board the research vessels (R/Vs) *Natsushima* and *Kaiyo* of Japan Agency for Marine‐Earth Science and Technology (JAMSTEC), or by the manned deep‐submergence vehicle *Jiaolong* on‐board the Chinese R/V *Xiangyanghong 9* (see Supporting Information Table S1 for details). The four hydrothermal vents included Dai‐yon Yonaguni Knoll (DK; 1,344 m depth) and Hatoma Knoll (HK; 1,482 m depth) in the southern OT (S‐OT), Iheya Ridge (IR; 1,402 m depth) and Iheya North (IN; two sites in 993 m and 1,002 m depth) in the middle OT (M‐OT); the two methane seeps included Jiaolong Ridge (JR; 1,122 m depth) in the SCS and Off Hatsushima (OH; two sites in 858 m and 1,172 m depth) in Sagami Bay (SB). These sampling locations span a horizontal distance of more than 2,400 km. Upon arrival on the deck, mussels were either dissected immediately for preservation in 95%–100% ethanol or frozen immediately at −80°C for later dissection. Genomic DNA was extracted from the adductor muscle of each individual using the phenol/chloroform extraction protocol (Sambrook, Fritsch, & Maniatis, 1989). Concentration and purity of the extracted DNA were measured using a NanoDrop ND‐1000 spectrophotometer (Thermo Fisher Scientific, Wilmington, DE, USA), and the integrity of DNA was checked by 1.0% agarose gel electrophoresis.

### 2.2 Mitochondrial gene amplification and genetic statistic estimation

The newly designed primer pair BP_*atp6*F (5′‐CATAGGAGCAAAGTAAGTGG‐3′) and BP_*atp6*R (5′‐GGTTCTACCACCATCCTCG‐3′), the universal primer pair LCO1490 and HCO2198 (Folmer, Black, Hoeh, Lutz, & Vrijenhoek, 1994), and the primer pair ArgBL and NAP2H (Arèvalo, Davis, & Sites, 1994; Bielawski & Gold, 1996) were used to amplify sequences of *atp6*,* cox1*, and *nad4* genes, respectively. Polymerase chain reactions (PCRs) were performed using a Mastercycler Gradient thermocycler (Eppendorf, Germany) with the following program: 2 min initial denaturation at 94°C, 30 cycles of 30 s denaturation at 94°C, 30 s annealing at 50°C, 30 s extension at 72°C, and a final extension for 10 min at 72°C. The PCR products were separated using 1.0% agarose gel electrophoresis and purified using the Zymoclean^™^ Gel DNA Recovery Kit (Zymo Research, Irvine, CA, USA) following the manufacturer's protocol. Purified PCR products were bidirectionally sequenced on an ABI PRISM^®^ 3730xl DNA Analyzer. Sequences obtained were visually checked and assembled into contigs using the DNASTAR Lasergene package (DNASTAR Inc., Madison, WI, USA). Each mitochondrial gene from all individuals was aligned using MUSCLE v.3.8.31 (Edgar, 2004) under the default settings and manually trimmed to the same length for subsequent analyses.

Pairwise sequence divergence was calculated based on the Kimura‐2‐parameter (K2P) model (Kimura, 1980) implemented in MEGA v.7 (Kumar, Stecher, & Tamura, 2016) for each mitochondrial gene. For individuals collected from each location (i.e., local population), the number of haplotypes (H), haplotype diversity (*Hd*), and nucleotide diversity (π) of each mitochondrial gene were estimated using Arlequin v.3.5.2.2 (Excoffier & Lischer, 2010). Two neutrality tests, including Tajima's *D* (Tajima, 1989) and Fu's *F*_S_ (Fu, 1997), implemented in Arlequin, were conducted on each mitochondrial gene of each local population, with 10,000 simulations performed to test for significance.

In addition, the three mitochondrial genes of each individual were concatenated into a single sequence using SequenceMatrix v.1.7.8 (Vaidya, Lohman, & Meier, 2011), and the concatenated sequences were then used to estimate pairwise *F*_ST_ between local populations using Arlequin, with 10,000 permutations applied to test for significance. Furthermore, to investigate the population structure, TCS haplotype networks were reconstructed for each mitochondrial gene using POPART v.1.7 (Leigh & Bryant, 2015).

### 2.3 RAD library construction, sequencing, and data filtering

The 2b‐RAD method (Wang, Meyer, McKay, & Matz, 2012) was applied to construct RAD libraries. Genomic DNA from each individual was digested using the type IIB restriction enzyme BsaXI (New England BioLabs, Ipswich, MA, USA). Two adaptors with compatible (5′‐NNN‐3′) overhangs were used to link the digested products, and a 6‐bp unique barcode was then added to each individual, resulting in DNA libraries of approximately 155 bp. These libraries were afterward purified using the QIAquick PCR Purification Kit (Qiagen, Chatsworth, CA, USA), analyzed for integrity by 8.0% polyacrylamide gel electrophoresis (PAGE), quantified using the Qubit 2.0 Fluorometer (Invitrogen, Carlsbad, CA, USA), and then pooled for single‐end sequencing on the Illumina HiSeq 1500, HiSeq 2000, and/or HiSeq 4000 platforms.

Raw sequencing reads were filtered using a custom Perl script to remove adaptors and the 3‐bp terminal sequences of each read (Jiao et al., 2014). Reads with ≥10 nucleotide positions having a Phred quality index <20, without restriction sites, with ambiguous bases, or ≥30% homopolymer regions, were all discarded.

### 2.4 SNP identification and genetic statistic estimation

Based on the recognition sites of the BsaXI enzyme, 2b‐RAD tags were extracted from the draft genome of *B. platifrons* (Sun et al., 2017), which served as a reference for SNP identification. Alignment of the filtered reads of each individual to the reference was achieved by SOAP v.2.21 (Li et al., 2009) using the match mode of “find the best hits” (‐M 4), the maximum number of allowed mismatches of two (‐v 2), and no repeat allowed (‐r 0). The output file for each individual was converted into the.sam format using soap2sam.pl (http://soap.genomics.org.cn/soapaligner.html).

Detection of SNPs was carried out using the ref_map.pl pipeline implemented in Stacks v.1.41 (Catchen, Hohenlohe, Bassham, Amores, & Cresko, 2013) with the following criteria: (a) ≥10 aligned reads to build a stack (‐m 10); (b) loci be biallelic as those with more alleles are likely caused by sequencing or clustering errors; (c) loci with a depth coverage (i.e., number of reads of a specific locus that matched to the reference) ≤120 to reduce bias derived from repetitive genomic contents; (d) loci present in all local populations and genotyped for ≥70% individuals in each local population; (e) SNPs with an overall minor allele frequency (MAF) ≥0.02 to reduce PCR and sequencing errors, as well as uninformative markers (Roesti, Salzburger, & Berner, 2012); (f) loci with an observed heterozygosity (*H*_obs_) ≤0.5 among all individuals to avoid inclusion of paralogs (Hohenlohe, Amish, Catchen, Allendorf, & Luikart, 2011); (g) SNPs conforming to Hardy–Weinberg equilibrium (*p *≥* *0.01) as assessed by the exact test implemented in Arlequin for each local population, with 100,000 dememorization steps followed by 1,000,000 steps in a Markov chain; (h) number of SNPs per locus ≤3.

Genomewide genetic statistics, including expected heterozygosity (*H*_exp_), *H*_obs_, π, and inbreeding coefficient (*F*_IS_), were calculate using Stacks. Two neutrality tests, including Tajima's *D* (Tajima, 1989) and Fu and Li's D* (Fu & Li, 1993), for each polymorphic loci of each local population, were estimated based on the batch mode implemented in DnaSP v.5 (Rozas, Sánchez‐DelBarrio, Messeguer, & Rozas, 2003).

Filtered SNP datasets were further formatted using the POPULATIONS module in Stacks, FORMATOMATIC v.0.8.1 (Manoukis, 2007), and/or PGDSpider v.2.0.8.3 (Lischer & Excoffier, 2012) for downstream analyses.

### 2.5 Outlier SNP detection and characterization

The coalescent method implemented in Arlequin was used to screen for candidate outlier SNPs. It has been reported that the hierarchical island model in this software is more powerful than the finite island model in outlier SNP detection for hierarchically subdivided populations or populations with a recent common ancestry (Excoffier, Hofer, & Foll, 2009). Therefore, based on the geographic affinity and the result of principal component analyses (PCA) carried out using the entire SNP dataset (see section 3.8), two hierarchical island models were assumed in Arlequin to screen for candidate outlier SNPs: (a) individuals were divided into two groups: SCS = JR, and the OT‐SB region = DK, HK, IR, IN, and OH; (b) individuals in JR and the three JR‐like individuals in the OH site of SB were excluded, and the remaining individuals were divided into three groups: S‐OT = DK and HK, M‐OT = IR and IN, and SB = OH. The outlier screening analyses were then carried out by running 100,000 simulations, along with 100 simulated demes and 50 stimulated groups.

Genomic regions of the identified candidate outlier SNPs were determined by mapping the 2b‐RAD tags that harbored outlier SNPs against the draft genome of *B. platifrons* (Sun et al., 2017). For those mapped to the genic regions [i.e., coding DNA sequence (CDS), intron, or 3/5′‐untranslated region (3/5′‐UTR)], their corresponding proteins (i.e., outlier‐associated proteins) and annotations (Sun et al., 2017) were extracted for functional classification.

### 2.6 Genetic differentiation estimation and its relatedness to geographic distance

Values of pairwise *F*_ST_ between local populations were estimated using Arlequin based on the entire SNP dataset and the two outlier SNP datasets, with 10,000 permutations to determine significance. The Mantel test implemented in the same software was carried out to correlate genetic distance (i.e., values of pairwise *F*_ST_ calculated based on the entire SNP dataset) and geographic distance (km), also with 10,000 permutations applied to test for significance. The approximate geographic distance between each pair of local populations was measured using the Latitude/Longitude Distance Calculator (http://jan.ucc.nau.edu/~cvm/latlongdist.html). An intermediate geographic point between the two sampling sites of IN and OH was separately generated to simplify the calculation.

### 2.7 Population structure and individual assignment based on the entire SNP dataset and the two outlier SNP datasets

A Bayesian approach implemented in STRUCTURE v.2.3.4 (Pritchard, Stephens, & Donnelly, 2000) was applied to detect population structure. The LOCPRIOR model which uses sampling locations as prior information to assist the clustering and the corrected allele frequencies model were selected. The number of genetic clusters *K* was set from 1 to 6, each with five replicates, using a burn‐in of 100,000 followed by 1,000,000 iterations. The optimal *K* was evaluated using STRUCTURE HARVESTER v.0.6.94 (Earl & vonHoldt, 2012). An optimal alignment of replicate runs at the optimal *K* was determined using CLUMPP v.1.1.2 (Jakobsson & Rosenberg, 2007), and the graph of genetic structure was visualized using DISTRUCT v.1.1 (Rosenberg, 2004). The PCA implemented in the R package SNPRelate (Zheng et al., 2012) were used to perform individual assignment based on the genetic variation among individuals.

All of the above analyses were carried out using both the entire SNP dataset and the two outlier SNP datasets separately, with only one SNP per locus retained in each dataset to avoid bias derived from potential linkage disequilibrium.

### 2.8 Introgression analyses based on the outlier SNP dataset

Since two genetic backgrounds were detected in the local population of HK based on the second outlier SNP dataset (see section 4.1), the USEPOPINFO model which uses sampling locations to screen for migrants or hybrids in STRUCTURE was applied to test the hypothesis of mixed ancestry for individuals in HK during the past two generations (i.e., GENSBACK = 2). Two clusters (i.e., *K *=* *2) were defined according to the results of STRUCTURE and PCA based on the second outlier SNP dataset (see section 4.1), with one containing individuals in the S‐OT, and the other containing those in the M‐OT and SB with the three JR‐like individuals in SB excluded. The program was run with a burn‐in of 100,000 followed by 1,000,000 iterations based on the second outlier SNP dataset (only one SNP per locus retained). Three different values of MIGRPRIOR, which is *v*, were applied to test whether the results were robust (Pritchard et al., 2000). Individuals with less than 50% posterior probability of having pure ancestry from the designated population (i.e., the S‐OT) were considered to be migrants or hybrid descendants (Falush, Stephens, & Pritchard, 2007; Pritchard et al., 2000).

Introgression signature in the local population of HK was further investigated by calculating the hybrid index (*h*) (Buerkle, 2005) using the R package INTROGRESS (Gompert & Alex Buerkle, 2010), also based on the second outlier SNP dataset (only one SNP per locus retained). According to the population structure revealed by STRUCTURE analyses based on the second outlier SNP dataset (see section 4.1), the local population of DK was designated as parental population 1, while those in the M‐OT and SB (the three JR‐like individuals in SB were excluded) were together designated as parental population 2. Values of *h* refer to the proportion of the genome of a given individual in the local population of HK that was inherited from the designated parental population 2 (Buerkle, 2005; Gompert & Alex Buerkle, 2010).

### 2.9 Migration dynamic analyses based on the entire SNP dataset

The web‐based software divMigrate‐online (Sundqvist, Keenan, Zackrisson, Prodöhl, & Kleinhans, 2016) was applied to infer the directional relative migration patterns using the *G*_ST_ statistic (Nei, 1973) as a measure of genetic differentiation. The method implemented in this software is based on defining a hypothetical pool of migrants for a given pair of populations and estimating an appropriate measure of genetic differentiation between each of the two populations and the hypothetical pool (Sundqvist et al., 2016). The directional genetic differentiation can be used afterward to evaluate the relative levels of migration between the two populations. The larger of the two relative migration values indicates the population is most likely the source population, whereas the smaller of the two values indicates the population is most likely to be the sink population (Sundqvist et al., 2016).

## 3 RESULTS

### 3.1 Population genetic analyses based on mitochondrial genes

**Figure 2:**
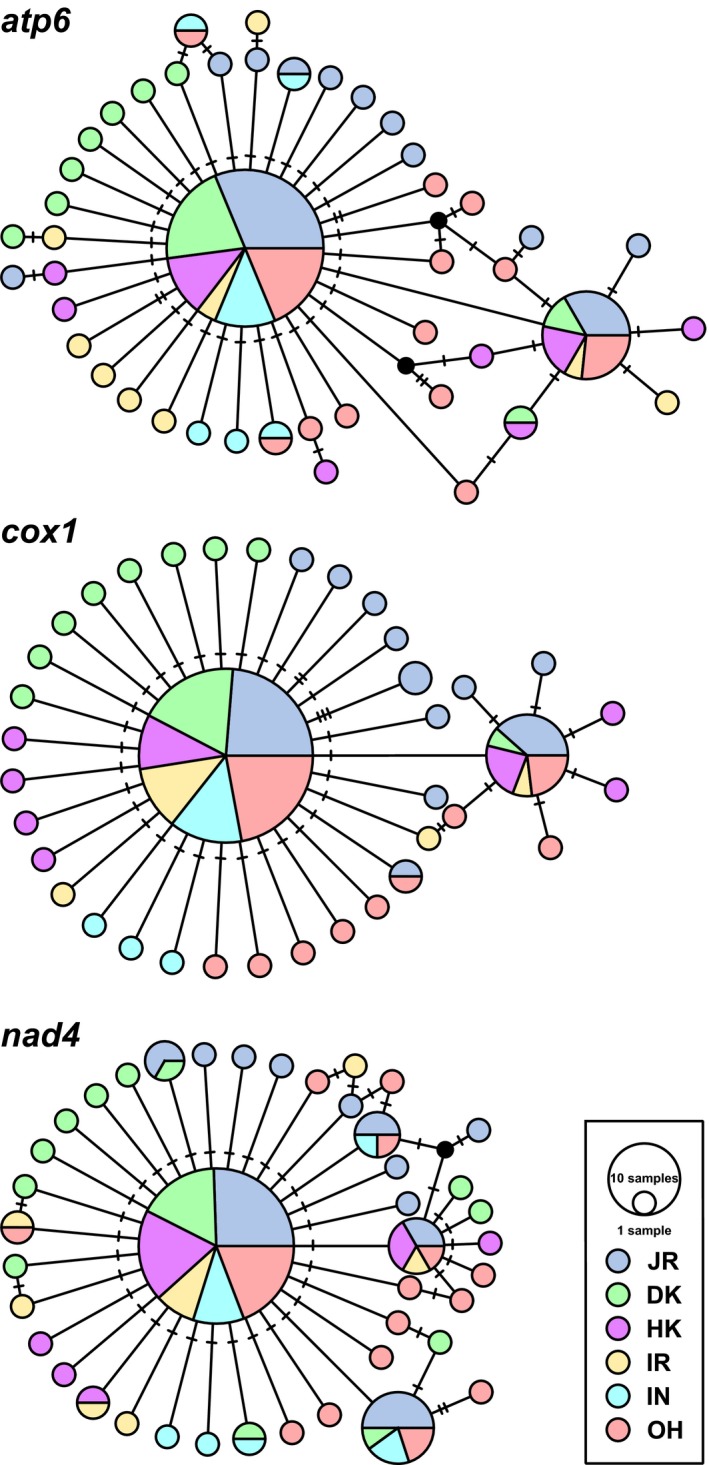
TCS haplotype networks inferred based on *atp6*,* cox1*, and *nad4*. The number of hatch marks along edges indicates the number of nucleotide substitutions. Color of each circle represents the sampling location where the haplotype was found, and size of each circle is proportional to the frequency of the respective haplotype. Black circles indicate unknown or missing haplotypes

Alignment and trimming of the amplified sequences resulted in 717‐bp full‐length *atp6*, 647‐bp partial *cox1*, and 597‐bp partial *nad4* gene sequences. Pairwise sequence divergence ranged from 0 to 0.84% (mean: 0.20%) for *atp6* (Supporting Information Table S2), 0 to 0.78% (mean: 0.17%) for *cox1* (Supporting Information Table S3), and 0 to 1.02% (mean: 0.22%) for *nad4* (Supporting Information Table S4). Values of *Hd* for each local population ranged from 0.7273 to 0.9778 for *atp6*, 0.4909 to 0.8286 for *cox1*, and 0.6476 to 0.8667 for *nad4*; values of π ranged from 0.0015 to 0.0030 for *atp6*, 0.0008 to 0.0022 for *cox1*, and 0.0015 to 0.0027 for *nad4* (Table 1). Most Tajima's *D* and all Fu's *F*_S_ statistics were significantly (*p *<* *0.05) negative for local populations of *B. platifrons* (Table 1). Values of pairwise *F*_ST_ calculated based on the three concatenated mitochondrial genes ranged from −0.0134 to 0.0782, with no statistical significance detected after Bonferroni correction (Table 2). TCS haplotype networks based on each mitochondrial gene all roughly exhibited a star‐like shape, with the most frequent haplotype shared among different locations in the center being surrounded by several low frequency and private haplotypes (Figure 2).

**Table 1:** Summary genetic statistics of each local population based on three mitochondrial genes

Gene	Location	*N*	*H*	*Hd*	π	Tajima's *D*	Fu's *F*_S_
*atp6* (717 bp)	JR	30	12	0.7356	0.0016	−2.1248**	−9.2918***
	DK	20	10	0.7579	0.0016	−2.0474**	−7.6,398***
	HK	15	8	0.8286	0.0020	−1.5158	−4.3642**
	IR	10	9	0.9778	0.0030	−1.7295*	−7.0432***
	IN	11	6	0.7273	0.0015	−1.8506*	−3.3039**
	OH	24	13	0.8478	0.0023	−2.0706**	−9.6958***
*cox1* (647 bp)	JR	30	12	0.7655	0.0022	−1.8726*	−7.8294***
	DK	20	10	0.7105	0.0014	−2.2189**	−9.3321***
	HK	15	8	0.8286	0.0020	−1.4664	−4.9120***
	IR	10	4	0.5333	0.0009	−1.5622*	−1.9637**
	IN	11	4	0.4909	0.0008	−1.6000*	−2.0423**
	OH	24	10	0.7065	0.0016	−1.9029*	−7.7136***
*nad4* (597 bp)	JR	30	12	0.8184	0.0021	−1.7635*	−8.7518***
	DK	20	13	0.8526	0.0025	−2.1633**	−12.1714***
	HK	15	6	0.6476	0.0015	−1.4512	−3.2353**
	IR	10	7	0.8667	0.0023	−1.8391**	−4.5227***
	IN	11	6	0.8000	0.0018	−1.4646	−3.4118**
	OH	24	15	0.8659	0.0027	−2.0000**	−14.2081***

Notes

π: nucleotide diversity; *H*: number of haplotypes; *Hd*: haplotype diversity; *N*: sample size.

Significance: *p <* *0.05; **p <* *0.01; ***p <* *0.001.

**Table 2:** Pairwise *F*_ST_ estimated based on the three concatenated mitochondrial genes (above diagonal) and the entire set of 6,398 SNPs (below diagonal)

	JR	DK	HK	IR	IN	OH
JR	—	0.0038	0.0052	0.0008	0.0103	−0.0097
DK	0.0201*	—	0.0255	−0.0124	−0.0087	−0.0041
HK	0.0188*	0.0013	—	0.0048	0.0782	−0.0014
IR	0.0162*	0.0032	−0.0008	—	0.0138	−0.0134
IN	0.0206*	0.0049	−0.0008	0.0004	—	0.0008
OH	0.0139*	0.0022	−0.0010	0.0008	−0.0005	—

Note

Significance: *p <* *0.00001 after Bonferroni correction.

### 3.2 SNP identification and genetic statistic estimation

Sequencing of 2b‐RAD libraries generated approximately 2.2 billion reads in total, with a mean of 19.9 million reads per individual. Quality control reduced the data to a mean of 15.5 million reads per individual, providing an average depth coverage of 46.3 × (Supporting Information Table S5). A total of 314,151 2b‐RAD tags were extracted from the 1.64 Gb genome (Sun et al., 2017), with one cutting site in every 5.2 kb. Among these tags, 177,365 (56.5%) were unique, 965 (0.3%) contained ambiguous bases (N), and 28,161 (43.2%) had repeats (repeat range: 2 to 1,378). All of them were used as the reference for subsequent SNP identification.

After genotyping and strict filtering, a total of 6,398 SNPs were identified from 5,458 2b‐RAD tags (Table 3; Supporting Information Table S6). The number of polymorphic nucleotide sites for the six local populations varied from 4,452 (accounting for 3.0% of the total nucleotide sites; IR) to 5,769 (accounting for 3.9% of the total nucleotide sites; JR). Since loci were retained only when they were detected in all local populations, only a few private SNPs were observed in each local population after filtering: 19 in JR, one in HK, three in OH, and none in DK, IR, or IN. Moreover, local populations of DK and HK in the S‐OT had lower values of *H*_obs_ and π as well as higher values of *F*_IS_ compared to those in the SCS, the M‐OT, and SB. All these statistics were summarized in Table 4.

**Table 3:** Number of the putative loci retained following each step of filtering and the final result of candidate SNPs

Category	Count
Putative loci after filtering steps	
Putative 2b‐RAD loci (stacks depth ≥10)	159,223
Polymorphic loci	91,487
Biallelic loci	76,317
Coverage depth ≤120	54,554
Present in all six populations and genotyped individuals in each population ≥70%	12,981
Overall minor allele frequency (MAF) ≥0.02	5,815
Observed heterozygosity (*H*_obs_) ≤0.5	5,661
Hardy–Weinberg equilibrium (*p *≥* *0.01)	5,464
Number of SNPs per locus ≤3	5,458
Entire candidate SNPs	
Total number	6,398
Number when one per locus was retained	5,458
Candidate outlier SNPs identified by Arlequin (*p *<* *0.01)	
SCS and OT‐SB	
Total number	106
Number when one per locus was retained	99
S‐OT, M‐OT, and SB	
Total number	138
Number when one per locus was retained	125

**Table 4:** Summary genetic statistics of each local population based on the entire set of 6,398 SNPs

Region	Location	Variant positions						
		Private	Variant sites	Poly sites	*H*_exp_	*H*_obs_	π	*F*_IS_
SCS	JR	19	6,398	5,769	0.1662	0.1620	0.1694	0.0292
S‐OT	DK	0	6,398	5,227	0.1553	0.1480	0.1594	0.0447
	HK	1	6,398	4,914	0.1586	0.1516	0.1648	0.0459
M‐OT	IR	0	6,398	4,452	0.1611	0.1635	0.1702	0.0193
	IN	0	6,398	4,656	0.1631	0.1631	0.1710	0.0246
SB	OH	3	6,398	5,683	0.1664	0.1633	0.1703	0.0278

Region	Location	All (variant and fixed) positions						
		Private	Total sites	% Poly	*H*_exp_	*H*_obs_	π	*F*_IS_
SCS	JR	19	147,366	3.9	0.0072	0.0070	0.0074	0.0013
S‐OT	DK	0	147,366	3.5	0.0067	0.0064	0.0069	0.0019
	HK	1	147,366	3.3	0.0069	0.0066	0.0072	0.0020
M‐OT	IR	0	147,366	3.0	0.0070	0.0071	0.0074	0.0008
	IN	0	147,366	3.2	0.0071	0.0071	0.0074	0.0011
SB	OH	3	147,366	3.9	0.0072	0.0071	0.0074	0.0012

% Poly: percentage of polymorphic sites in total nucleotide sites (i.e., total sites); *F*_IS_: inbreeding coefficient; *H*_exp_: expected heterozygosity; *H*_obs_: observed heterozygosity; Poly sites: number of polymorphic sites; Private: number of unique SNPs; π: nucleotide diversity.

Total sites: 27 nucleotide sites per locus × 5,458 loci = 147,366 nucleotide sites.

No polymorphic loci were detected to have a significantly negative value of Tajima's *D* in any local populations, whereas a very small proportion of polymorphic loci (i.e., <0.4%) were detected to have a significantly (*p *<* *0.05) positive value of Tajima's *D* in each local population (Supporting Information Table S7). Besides, less than 1.1% of polymorphic loci were detected to have a significantly (*p *<* *0.05) negative value of Fu and Li's D* in the local populations of JR, DK, HK, and OH; nevertheless, no polymorphic loci were detected to have a value of Fu and Li's D* with statistical significance in the local population of IR or IN (Supporting Information Table S8).

### 3.3 Genetic differentiation calculated based on the entire SNP dataset and its relatedness to geographic distance

Values of pairwise *F*_ST_ calculated based on the entire set of 6,398 SNPs ranged from −0.0010 to 0.0206, with statistical significance (*p *<* *0.00001) detected only between the local population of JR and all the others in the OT‐SB region (*F*_ST_ range: 0.0139 to 0.0206) after Bonferroni correction (Table 2).

The Mantel test revealed no correlation between genetic distance represented by values of pairwise *F*_ST_ calculated based on the entire set of 6,398 SNPs and the geographic distance (Supporting Information Figure S1), showing no evidence for isolation‐by‐distance throughout the known distribution range of *B. platifrons*.

### 3.4 Population structure and individual assignment based on the entire SNP dataset

**Figure 3:**
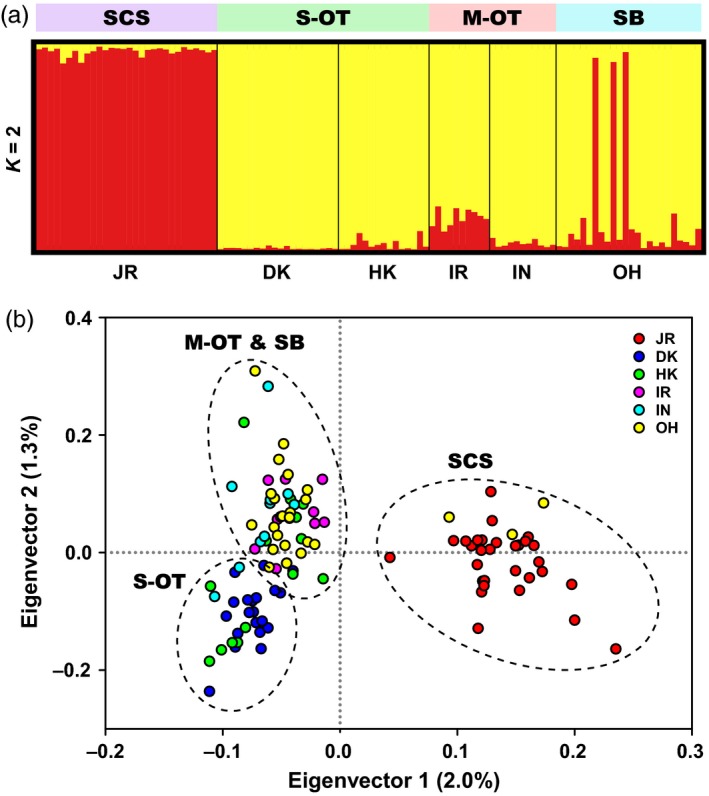
Population genetic structure of *B. platifrons* inferred based on the entire set of 5,458 SNPs (only one SNP per locus retained) using (a) STRUCTURE analyses and (b) PCA implemented in SNPRelate. In (a), each individual is represented by a single bar, with different colors showing membership fractions of each inferred cluster

STRUCTURE analyses based on the entire set of 5,458 SNPs (only one SNP per locus retained) revealed the occurrence of two genetic groups (i.e., optimal *K *=* *2) of *B. platifrons* in the Northwest Pacific (Supporting Information Figure S2a,b). One genetic group consisted of all individuals in JR of the SCS as well as three individuals in OH (i.e., OH1_4, OH1_7, and OH1_9) of SB, and the other genetic group was formed by the remaining individuals in the OT‐SB region (Figure 3a). This pattern of population genetic structure was also detected in the result of PCA along the first eigenvector (Figure 3b). In addition, there was a minor genetic subdivision between most individuals in the S‐OT and those in the M‐OT and SB along the second eigenvector (Figure 3b).

### 3.5 Identified outlier SNPs based on two hierarchical island models

**Figure 4:**
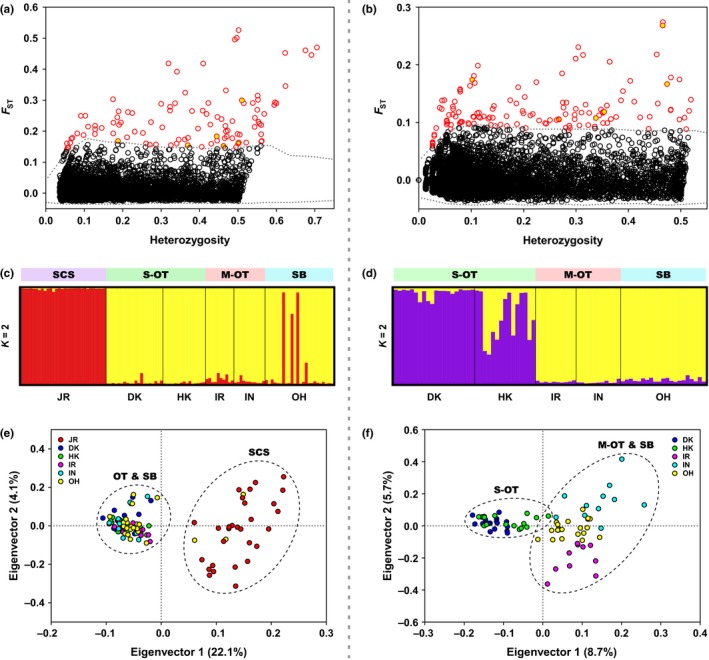
Population genetic structure of *B. platifrons* inferred based on the two outlier SNP datasets. Locus‐specific *F*_ST_ is plotted against observed heterozygosity (Heterozygosity) with red circles indicating (a) 106 outlier SNPs under the hierarchical island model: SCS= JR and the OT‐SB region= DK, HK, IR, IN, and OH, and (b) 138 outlier SNPs identified using Arlequin under the hierarchical island model: S‐OT = DK and HK, M‐OT = IR and IN, and SB = OH with the three JR‐like individuals in the OH site of SB excluded. Yellow filled circles represent the seven outliers found in both assumed models. Results of STRUCTURE analyse (c, d) and PCA (e, f) based on the outlier SNPs identified in (a) and (b), respectively. Only one SNP per locus in each dataset, that is, 99 outlier SNPs identified in (a) and 125 outlier SNPs identified in (b), was retained for STRUCTURE analyses and PCA to avoid bias derived from potential linkage disequilibrium

By defining a hierarchical island model composed of the SCS and the OT‐SB region in Arlequin, a total of 106 candidate outlier SNPs associated with 99 loci (*p *<* *0.01) were identified (Figure 4a), with the allele frequency of each outlier SNP shown in Supporting Information Table S9. Among them, 32 outlier SNPs associated with 30 loci were mapped to 27 proteins derived from the draft genome of *B. platifrons* (Sun et al., 2017). These outliers were found in different regions throughout the genome, including six (5.7%) in CDSs with two being nonsynonymous substitutions, 20 (18.9%) in introns, and six (5.7%) in 3′‐UTRs. The outlier‐associated proteins were manually classified into ten broad categories, including biological adhesion (2, 7.4%), carbohydrate and lipid metabolism (2, 7.4%), cell death (1, 3.7%), DNA metabolism (4, 14.8%), localization (4, 14.8%), peptide metabolism (1, 3.7%), response to stimulus (3, 11.1%), signaling (3, 11.1%), system development and processing (2, 7.4%), and those with unknown functions (5, 18.6%) (Supporting Information Table S10).

By defining a hierarchical island model composed of the S‐OT, the M‐OT, and SB, a total of 138 candidate outlier SNPs associated with 125 loci (*p *<* *0.01) were identified (Figure 4b), with the allele frequency of each outlier SNP shown in Supporting Information Table S11. A total of 46 outlier SNPs associated with 39 loci were mapped to 38 proteins derived from the draft genome of *B. platifrons* (Sun et al., 2017). Among these outliers, 11 (8.0%) were found in CDSs with five being nonsynonymous substitutions, 28 (20.3%) in introns, and seven (5.1%) in 3′‐UTRs. The outlier‐associated proteins were manually classified into ten broad categories, including carbohydrate (derivative) and lipid metabolism (3, 7.9%), cell death (1, 2.6%), DNA metabolism (3, 7.9%), localization (6, 15.8%), protein folding, assembly, and metabolism (3, 7.9%), response to stimulus (3, 7.9%), signaling (6, 15.8%), system development and processing (4, 10.5%), transcription and translation (4, 10.5%), as well as those with unknown functions (5, 13.2%) (Supporting Information Table S12).

Although the number of candidate outliers was comparable under the two assumed hierarchical island models, only seven SNPs were shared between these two outlier SNP datasets (Figure 4a,b), indicating the two hierarchical island models relied on different genetic architecture.

### 3.6 Genetic differentiation calculated based on the two outlier SNP datasets

Values of pairwise *F*_ST_ calculated based on the first outlier SNP dataset containing 106 outlier SNPs ranged from −0.0099 to 0.2785, with statistical significance (*p *<* *0.00001) detected in all pairwise evaluations between the local population of JR and those in the OT‐SB region (*F*_ST_ range: 0.1953 to 0.2785) after Bonferroni correction (Supporting Information Table S13).

Values of pairwise *F*_ST_ calculated based on the second outlier SNP dataset containing 138 outlier SNPs ranged from 0.0417 to 0.1964, with statistical significance (*p *<* *0.00001) detected in all pairwise estimations between local populations in the OT‐SB region after Bonferroni correction (Supporting Information Table S14). Among them, values of pairwise *F*_ST_ calculated between the two local populations in the S‐OT (*F*_ST_ = 0.0685) as well as those calculated between pairs of the three local populations in the M‐OT and SB (*F*_ST_ range: 0.0417 to 0.0778) were smaller (Supporting Information Table S14). In contrast, values of pairwise *F*_ST_ calculated between the local populations in the S‐OT and those in the M‐OT or SB (*F*_ST_ range: 0.0799 to 0.1964) were larger (Supporting Information Table S14).

### 3.7 Population structure and individual assignment based on the two outlier SNP datasets

STRUCTURE analyses based on the first outlier SNP dataset containing 99 outlier SNPs (only one SNP per locus retained) revealed two genetic groups (i.e., optimal *K *=* *2) of *B. platifrons* in the Northwest Pacific (Supporting Information Figure S2c,d; Figure 4c), which was in agreement with not only the result of PCA based on the first outlier SNP dataset containing 99 outlier SNPs (only one SNP per locus retained) along the first eigenvector (Figure 4e), but also the result of STRUCTURE analyses based on the entire set of 5,458 SNPs (only one SNP per locus retained) (Figure 3a).

STRUCTURE analyses based on the second outlier SNP dataset containing 125 outlier SNPs (only one SNP per locus retained) uncovered two genetic groups (i.e., optimal *K *=* *2) of *B. platifrons* in the OT‐SB region (Supporting Information Figure S2e,f), with one chiefly composed of individuals in the S‐OT and the other mainly composed of those in the M‐OT and SB (Figure 4d). However, two genetic backgrounds were detected in the local population of HK in the S‐OT (Figure 4d). When using these outlier SNPs for PCA, all individuals in the S‐OT were clustered together and formed a separate genetic group from those in the M‐OT and SB along the first eigenvector (Figure 4f). Additionally, individuals in IR, IN, and OH appeared to form three small genetic groups along the second eigenvector (Figure 4f), which was also observed in STRUCTURE analyses when forcing *K *=* *4 (Supporting Information Figure S3).

### 3.8 Signature of introgression in HK based on the outlier SNP dataset

By using the USEPOPINFO model in STRUCTURE analyses based on the second outlier SNP dataset containing 125 outlier SNPs (only one SNP per locus retained), eight individuals (53.3%) in the local population of HK were identified to have mixed ancestry as either migrants or hybrid descendants (Table 5). The consistency of results based on different *v* values indicated the estimation to be robust.

**Table 5:** Ancestry inference for *B. platifrons* in the local population of HK inferred by STRUCTURE based on the second outlier SNP dataset containing 125 outlier SNPs (only one SNP per locus retained). We estimated the posterior probabilities (*q*) under three MIGRPRIOR values (*v*) for each individual to have ancestry in the population group formed by individuals in the S‐OT (i.e., *q* prior pop), or in the population group formed by those in the M‐OT and SB in the present generation (present), in the first past generation (parent), or the second past generation (grandparent). Individuals in bold indicate those which can be considered as migrants or hybrid descendants

Individual	Prior pop	*v*	*q* prior pop	*q* M‐OT and SB		
				Present	Parent	Grandparent
HK_1	S‐OT	0.01	1	0	0	0
		0.05	1	0	0	0
		0.1	1	0	0	0
HK_10	S‐OT	0.01	1	0	0	0
		0.05	1	0	0	0
		0.1	1	0	0	0
**HK_11**	S‐OT	0.01	0	0.988	0.011	0
		0.05	0	0.994	0.006	0
		0.1	0	0.996	0.004	0
**HK_12**	S‐OT	0.01	0	0.737	0.26	0.003
		0.05	0	0.834	0.165	0.002
		0.1	0	0.883	0.116	0.001
**HK_13**	S‐OT	0.01	0	0.073	0.441	0.485
		0.05	0	0.113	0.458	0.429
		0.1	0	0.176	0.444	0.381
**HK_14**	S‐OT	0.01	0	0.061	0.811	0.128
		0.05	0	0.082	0.814	0.104
		0.1	0	0.104	0.807	0.089
**HK_15**	S‐OT	0.01	0.046	0	0.295	0.659
		0.05	0.005	0	0.348	0.647
		0.1	0.001	0	0.384	0.615
HK_2	S‐OT	0.01	0.997	0	0	0.002
		0.05	0.987	0	0.001	0.011
		0.1	0.973	0	0.003	0.024
HK_3	S‐OT	0.01	1	0	0	0
		0.05	0.999	0	0	0.001
		0.1	0.997	0	0	0.003
**HK_4**	S‐OT	0.01	0	0.094	0.708	0.198
		0.05	0	0.118	0.709	0.173
		0.1	0	0.132	0.708	0.16
HK_5	S‐OT	0.01	0.977	0	0	0.023
		0.05	0.852	0	0	0.147
		0.1	0.684	0	0.001	0.315
HK_6	S‐OT	0.01	1	0	0	0
		0.05	0.999	0	0	0.001
		0.1	0.998	0	0	0.002
HK_7	S‐OT	0.01	1	0	0	0
		0.05	0.999	0	0	0.001
		0.1	0.999	0	0	0.001
**HK_8**	S‐OT	0.01	0	0.486	0.401	0.113
		0.05	0	0.57	0.355	0.076
		0.1	0	0.636	0.306	0.058
**HK_9**	S‐OT	0.01	0.188	0	0.075	0.738
		0.05	0.022	0	0.098	0.879
		0.1	0.006	0	0.107	0.887

Values of *h* for each individual in the local population of HK computed by INTROGRESS based on the second outlier SNP dataset containing 125 outlier SNPs (only one SNP per locus retained) ranged from 0.0373 to 0.7033 (Table 6). Among them, the eight individuals showing mixed ancestry revealed by STRUCTURE analyses were estimated to have a moderate to large values of *h* ranging from 0.3643 to 0.7033, which indicated that these individuals were more likely hybrid descendants rather than migrants.

**Table 6:** The hybrid index (*h*) computed by INTROGRESS for *B. platifrons* in the local population of HK based on the second outlier SNP dataset containing 125 outlier SNPs (only one SNP per locus retained)

Individual	*h*	95% CI
HK_1	0.0373	(0.0020, 0.1624)
HK_10	0.1006	(0.0173, 0.2797)
**HK_11**	0.6874	(0.4703, 0.9172)
**HK_12**	0.7033	(0.4607, 0.9454)
**HK_13**	0.5302	(0.3161, 0.783)
**HK_14**	0.6013	(0.3712, 0.8596)
**HK_15**	0.4886	(0.2734, 0.7555)
HK_2	0.3591	(0.0642, 0.8843)
HK_3	0.1629	(0.0407, 0.3972)
**HK_4**	0.5987	(0.3719, 0.8548)
HK_5	0.2217	(0.0746, 0.4476)
HK_6	0.0995	(0.0171, 0.2776)
HK_7	0.0916	(0.0150, 0.2699)
**HK_8**	0.6574	(0.4292, 0.9071)
**HK_9**	0.3643	(0.1588, 0.6334)

CI: confidence interval.

Individuals in bold indicate those which can be considered as migrants or hybrid descendants by using STRUCTURE.

### 3.9 Directional relative migration patterns inferred based on the entire SNP dataset

**Figure 5:**
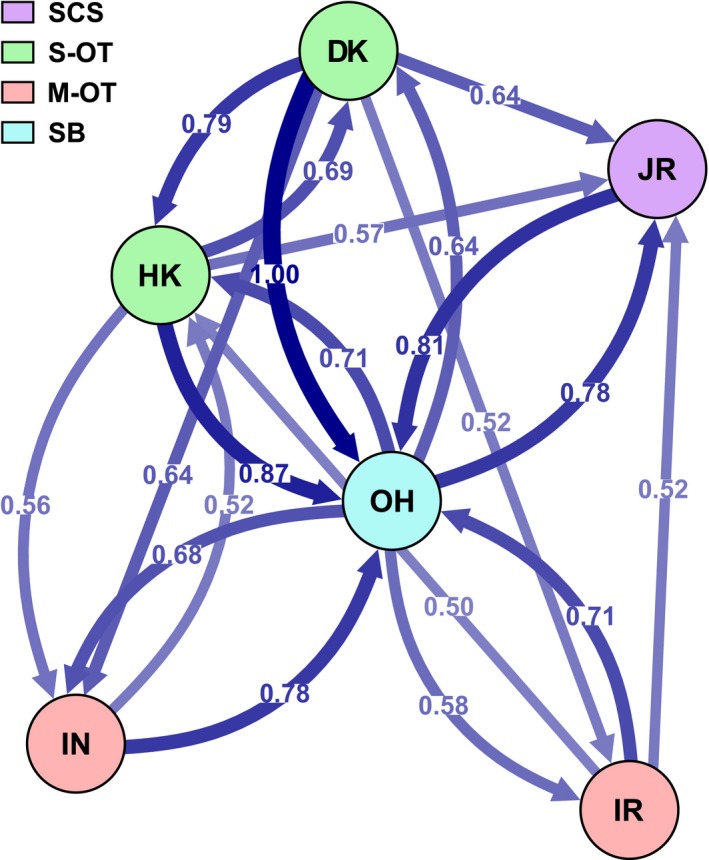
Directional relative migration of *B. platifrons* estimated using divMigrate‐online based on the entire set of 5,458 SNPs (only one SNP per locus retained) between sampling locations. Arrows refer to the direction of gene flow. Different colors of the circles indicate the geological regions of the sampling locations. Arrows with larger numbers display thicker in shape and darker in color. Numbers on the arrows represent the relative migration coefficients derived from *G*_ST_ statistics. Only the coefficients larger than 0.50 are indicated, and the complete relative directional migration matrix is shown in Supporting Information Table S15

Migration dynamic analyses based on the entire set of 5,458 SNPs (only one SNP per locus retained) revealed extensive gene flow between local populations of *B. platifrons*, especially for those in the OT‐SB region (Figure 5, Supporting Information Table S15). Although a higher level of gene flow was detected between the local populations of JR and OH, gene flow between the local population of JR and the others in the OT was found to be limited. Furthermore, gene flow from the local population of DK in the S‐OT to the others in the SCS, the M‐OT, and SB was stronger than that in the reverse directions.

## 4 DISCUSSION

Mitochondrial genes and genomewide SNPs were both applied in the present study with the aim to have a deeper understanding of the population genetics of the deep‐sea mussel *B. platifrons* in the Northwest Pacific. By using the three concatenated mitochondrial genes, no significant genetic differentiation was detected between any pairs of the six local populations. Meanwhile, the haplotype network constructed based on each mitochondrial gene also revealed no obvious genetic structure. These results were consistent with previous studies based on mitochondrial markers, indicating a high dispersal capability and a lack of population differentiation of *B. platifrons* in the Northwest Pacific (Kyuno et al., 2009; Miyazaki et al., 2013; Shen et al., 2016).

Analyses using the entire SNP dataset also indicated a high level of gene flow in *B. platifrons* manifesting by the small pairwise *F*_ST_ values and the result of migration dynamic analyses. Nevertheless, by using the entire SNP dataset and the first outlier SNP dataset, significant genetic differentiation was uncovered between the local population in the SCS and those in the OT‐SB region, and a clear genetic subdivision was detected between individuals in the SCS and those in the OT‐SB region. Moreover, by using the entire SNP dataset and the second outlier SNP dataset, a minor genetic divergence was uncovered between individuals in the S‐OT and those in the M‐OT and SB.

The discrepancy between results obtained from analyzing mitochondrial genes and SNPs was considered to be due to the different number and types of genetic markers used, resulting in different resolution. It has been reported that analyses of only a single or a few genetic markers can lead to relatively larger confidence intervals for very small values of *F*_ST_, which may result in non‐statistical significance for species with extensive gene flow (Blanco‐Bercial & Bucklin, 2016). Furthermore, it has been shown that selective sweeps at mitochondrial loci might be more common for organisms living in chemosynthesis‐based ecosystems in the deep ocean when compared with those in other marine environments (Roterman, Copley, Linse, Tyler, & Rogers, 2016). The significantly negative values of Tajima's *D* and Fu's *F*_S_ statistics estimated based on the mitochondrial genes of *B. platifrons* indicated a signature of selective sweeps at these mitochondrial genes or population expansion after a recent bottleneck (Fu, 1997; Tajima, 1989). However, the dual analyses of Tajima's *D* and Fu and Li's D* statistics on SNP markers paved an argument for selective sweeps at the mitochondrial genes, as only a few SNP markers agreed with the scenario of population expansion. In addition, the scenario of selective sweeps at the mitochondrial genes also fitted well with the observed lack of genetic differentiation between local populations of *B. platifrons* as revealed by the mitochondrial genes. Therefore, we focused our discussion below on the results obtained by using genomewide SNPs.

### 4.1 Co‐occurrence of two cryptic semi‐isolated lineages of *B. platifrons* in the Northwest Pacific

Oceanographic connection between the semi‐enclosed marginal SCS and the Northwest Pacific is mainly achieved through the seasonal intrusions of the Kuroshio Current, the North Pacific Intermediate Water (NPIW), as well as the Pacific Deep Water through the Luzon Strait (Nan et al., 2011; Qu, Girton, & Whitehead, 2006; You et al., 2005). The Kuroshio Current is a dominant and strong warm surface current in the Northwest Pacific that originates from the North Equatorial Current and runs northeastward along the Philippines coast (Andres et al., 2015). When passing by the Luzon Strait, a branch of the Kuroshio water can intrude into the SCS in a strong anticyclonic circulation, which is known as the “looping path,” and then flows out of the SCS to join its mainstream that continues to run toward the eastern Taiwan, the outer shelf of the East China Sea, passing by SB, and extending to the southeastern Japan (Figure 1b; Andres et al., 2015; Nan et al., 2011). The NPIW is a mid‐depth water mass widely distributed in the North Pacific subtropical gyre, which can flow from the northeastern part of North Pacific into the SCS, especially during winter and spring (You et al., 2005). These ocean currents make it possible for individuals of *B. platifrons* on the two sides of the Luzon Strait to exchange larvae. However, as revealed by the result of migration dynamic analyses, such exchange tended to be limited due to the relatively small volume of seawater involved. Therefore, the Luzon Strait may have served as a dispersal barrier, either promoting the formation of two cryptic semi‐isolated lineages of *B. platifrons* (i.e., the JR lineage and the OT‐SB lineage) in the Northwest Pacific or trapping a preexisting genetic barrier. Similar situations have also been reported from other *Bathymodiolus* mussels, such as those from the mid‐Atlantic Ridge (e.g., Breusing, Vrijenhoek, & Reusch, 2017; Breusing et al., 2016; Faure, Jollivet, Tanguy, Bonhomme, & Bierne, 2009; O'Mullan, Maas, Lutz, & Vrijenhoek, 2001) and the East Pacific (e.g., Plouviez et al., 2013).

Intriguingly, the results of STRUCTURE analyses and PCA revealed that three individuals in the OH site of SB had an extremely high genetic similarity to those dominating the JR site in the SCS, despite no such individuals being found in any sampled vent fields in the OT that lies between SB and the SCS. This phenomenon might be a consequence of long‐distance migration events between JR and OH as revealed by the result of migration dynamic analyses. Another hypothesis is that the two cryptic semi‐isolated lineages of *B. platifrons* may have a difference in habitat preference as it has been observed in coastal mussels (Bierne, Bonhomme, & David, 2003), with the lineage dominating JR preferring methane seeps. Nevertheless, broader sampling is required in the future to better test this hypothesis.

### 4.2 Genetic homogeneity, fine‐scale genetic structure, and admixture of *B. platifrons* in the OT‐SB region

The absence of genetic differentiation for *B. platifrons* in the OT‐SB region was revealed by the nonsignificant pairwise *F*_ST_ values and the result of STRUCTURE analyses based on the entire SNP dataset, indicating that the mainstream of the Kuroshio Current and the NPIW have played a vital role in promoting their larval dispersal in this area. Nevertheless, carrying out the above analyses and PCA using the second outlier SNP dataset, individuals in the S‐OT were detected to be genetically separated from those in the M‐OT and SB. Such fine‐scale population genetic structure could be explained as a result of natural selection in response to local adaptation (Gagnaire et al., 2015; Milano et al., 2014). Furthermore, the topography of the OT may have also played a key role in shaping the observed population subdivision. The OT is a back‐arc rifting basin formed behind the Ryukyu trench‐arc system. The M‐OT is located at the transitional region between the deeper S‐OT and the shallower northern OT, which is associated with the occurrence of intra‐trough grabens (Ikegami, Tsuji, Kumagai, Ishibashi, & Takai, 2015). These geological settings indicate the existence of subtle geographic barriers between the S‐OT and the M‐OT, which may decrease the dispersal success of *B. platifrons* larvae across different regions of the OT.

In addition, the ancestry inference of STRUCTURE analyses and the moderate to large values of *h* revealed that eight individuals in the local population of HK to be hybrid descendants of individuals in the S‐OT mating with those originating from the M‐OT and SB. This result indicated that HK may represent a contact zone for larval dispersal and genetic exchange of *B. platifrons* in the OT‐SB region. Individuals of *B. platifrons* in HK inhabited the deepest vent field included in this study, and this vent field is contained within a caldera. Therefore, it is possible that the introgression signature observed here was related to such bathymetry and topography, which may serve to trap mussel larvae from different genetic groups.

### 4.3 Directional migration patterns of *B. platifrons* in the Northwest Pacific

The result of migration dynamic analyses revealed that gene flow of *B. platifrons* in the Northwest Pacific to be asymmetrical, in that the gene flow was stronger from the S‐OT (especially DK) toward the SCS, the M‐OT, and SB than that in the reverse directions. Such migration patterns indicated that the local populations in the S‐OT tended to be the source populations of *B. platifrons* in the Northwest Pacific, which was in agreement with the lower values of *H*_obs_ and π as well as higher values of *F*_IS_ exhibited by the local populations in the S‐OT compared to those of the other local populations. However, this deduction should be treated with caution, since additional populations of *B. platifrons* elsewhere in the Northwest Pacific likely remain unsampled.

## 5 CONCLUSIONS AND PERSPECTIVES

By using genomewide SNPs rather than mitochondrial genes, two cryptic semi‐isolated lineages of *B. platifrons* in the Northwest Pacific were identified in this study, which may have been formed due to the barrier effect of the Luzon Strait or the contact zone been trapped by it. Among them, one lineage is mainly distributed in the semi‐enclosed marginal SCS, while the other is mainly distributed across the OT‐SB region. In addition, a fine‐scale population structure was detected for *B. platifrons* in the OT‐SB region, which might be due to the existence of subtle geographic barriers between the S‐OT and the M‐OT. The occurrence of three individuals with a high genetic affinity to those in JR in the OH site of SB remains puzzling and warrants further investigation to test whether this is related to differences in habitat preferences (i.e., methane seeps vs hydrothermal vents). The mixed genetic backgrounds detected in the local population of HK in the S‐OT indicated that this area may represent a contact zone for larvae from different locations in the OT‐SB region. Moreover, the local populations in the S‐OT (especially DK) may serve as a potential source of *B. platifrons* in the Northwest Pacific.

Overall, the present study has enhanced our understanding of the genetic connectivity, fine‐scale genetic structure, and migration patterns of *B. platifrons*. Together with several recent studies (Breusing et al., 2016, 2017), this study exemplified the usefulness of SNP data especially from high‐throughput sequencing for population genetic studies of chemosynthetic ecosystems. More importantly, the results from this study will lay a foundation for an effective determination of biogeographic regions, establishment of informed management plans, and designation of deep‐sea reserves in the Pacific Ocean, in preparation for an upcoming era of deep‐sea resource exploitation (Miller et al., 2018; Sigwart, Chen, & Marsh, 2017).

## DATA ARCHIVING STATEMENT

The mitochondrial gene sequences were deposited in GenBank of National Center for Biotechnology Information (NCBI) under the accession numbers of MH389991 to MH390100 for *cox1*, MH390101 to MH390210 for *nad4*, and MH390211 to MH390320 for *atp6*. Raw reads of 2b‐RAD libraries were deposited in the Sequence Read Archive (SRA) database of NCBI under the accession number of SRP149310.

## AUTHORS’ CONTRIBUTIONS

JWQ, PYQ, TX, and JS conceived this project. JWQ, JS, HKW, CC, MN, and DF collected the samples. TX extracted DNA, conducted 2b‐RAD libraries under the guidance of JL, SW, and ZB, performed bioinformatics analyses, and drafted the manuscript. RJ helped interpret the roles of ocean currents in population connectivity among mussel populations. All authors contributed to improvement of the manuscript.
