# Supplementary figures and images for: Population genetic structure of the deep‐sea mussel Bathymodiolus platifrons (Bivalvia: Mytilidae) in the Northwest Pacific
Source: Evol Appl. 2018 Oct 12;11(10):1915–30. doi: 10.1111/eva.12696 (PMC6231483; doi:10.1111/eva.12696)

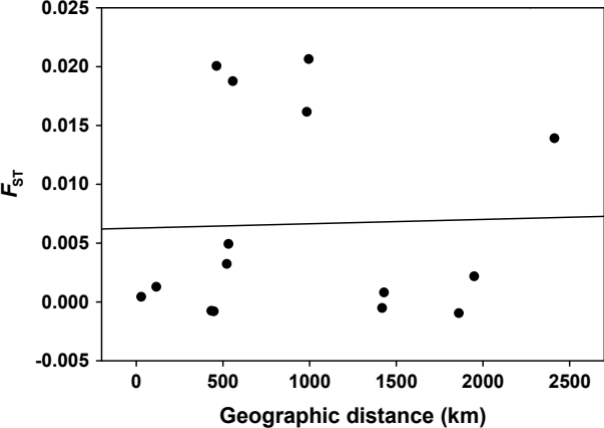

Supplement: Supplementary file 1 [file EVA-11-1915-s001.pdf]

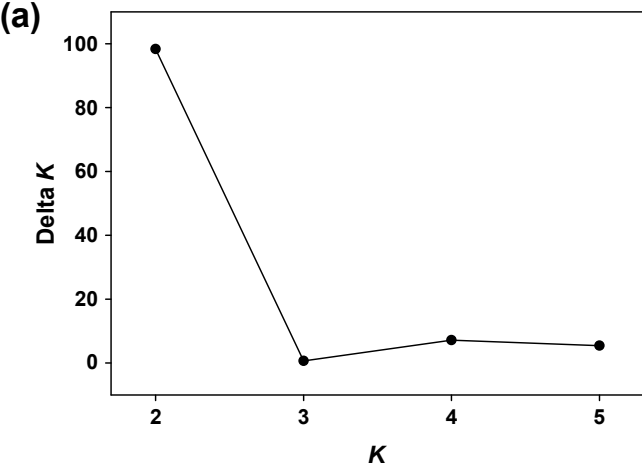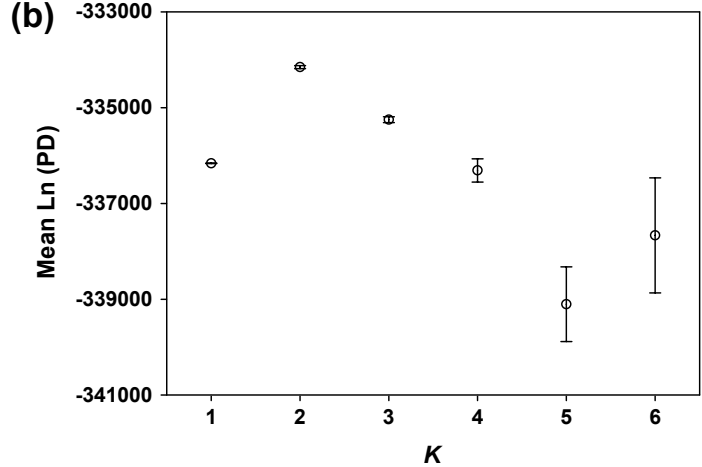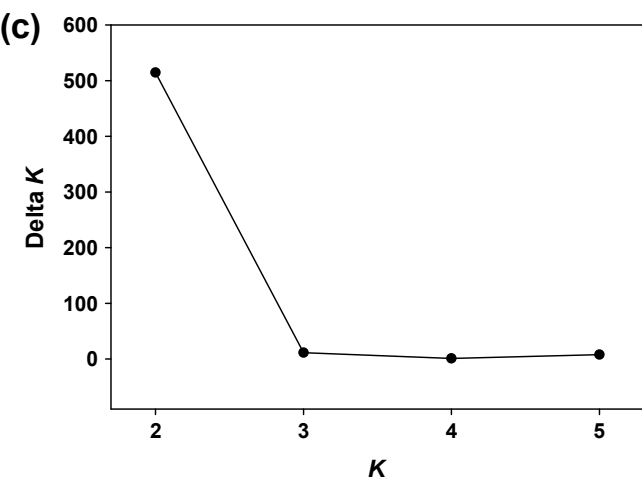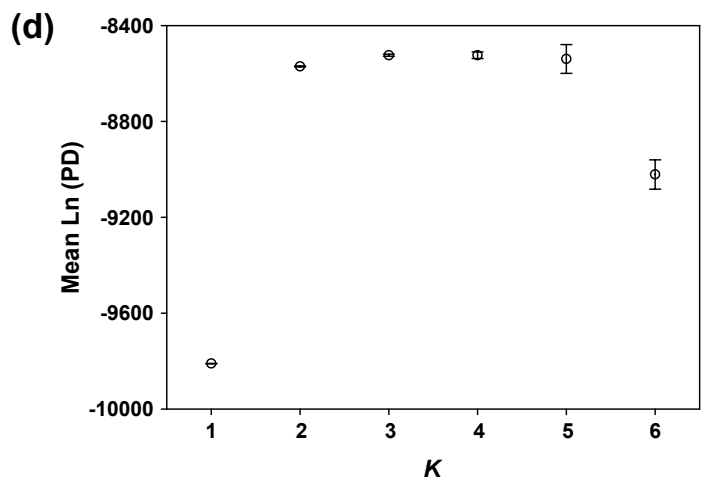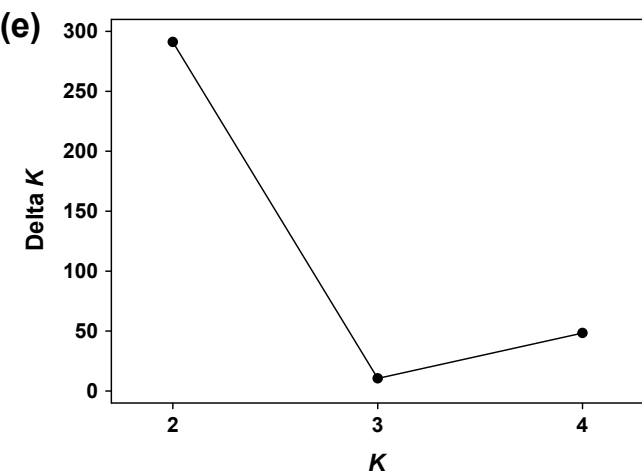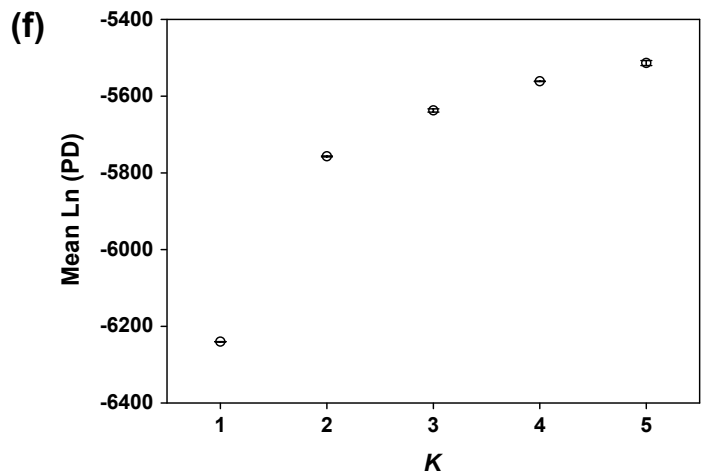

Supplement: Supplementary file 2 [file EVA-11-1915-s002.pdf]

**S-OT****M-OT****SB** $K=3$ 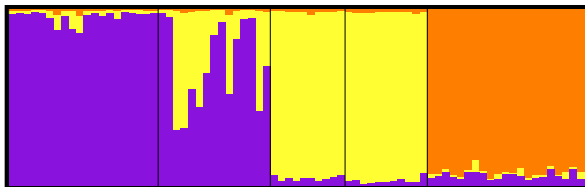 $K=4$ 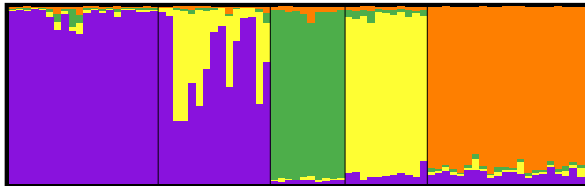

DK

HK

IR

IN

OH

Supplement: Supplementary file 3 [file EVA-11-1915-s003.pdf]
