# Supplementary material for: Population genetic structure of the deep‐sea mussel Bathymodiolus platifrons (Bivalvia: Mytilidae) in the Northwest Pacific
Source: Evol Appl. 2018 Oct 12;11(10):1915–30. doi: 10.1111/eva.12696 (PMC6231483; doi:10.1111/eva.12696)
Supplement: Supplementary file 5 [file EVA-11-1915-s005.docx]

**SUPPLEMENTARY FIGURE LEGENDS**

**FIGURE S1** Pairwise genetic distance (*F*_ST_) calculated based on the entire set of 6,398 SNPs plotted against geographic distance (km) between sampling locations, indicating barely no relatedness between genetic and geographic distance of *B. platifrons*.

**FIGURE S2** Estimation of the optimal *K* for STRUCTURE analyses to interpret population genetic structure of *B. platifrons* using STRUCTURE HARVESTER. Delta *K* and Mean Ln (PD) plotted against each *K* for STRUCTURE analyses based on (a, b) the entire set of 5,458 SNPs, (c, d) the first outlier SNP dataset containing 99 outlier SNPs, and (e, f) the second outlier SNP dataset containing 125 outlier SNPs. Only one SNP per locus in each dataset was retained to avoid bias derived from potential linkage disequilibrium.

**FIGURE S3** Population genetic structure of *B. platifrons* inferred based on the second outlier SNP dataset containing 125 outlier SNPs (only one SNP per locus retained) using STRUCTURE analyse when forcing *K* = 3 and *K* = 4. Each individual is represented by a single bar, with different colors showing membership fractions of each inferred cluster.
